# Task-Dependent Inhomogeneous Muscle Activities within the Bi-Articular Human Rectus Femoris Muscle

**DOI:** 10.1371/journal.pone.0034269

**Published:** 2012-03-27

**Authors:** Naokazu Miyamoto, Taku Wakahara, Yasuo Kawakami

**Affiliations:** Faculty of Sport Sciences, Waseda University, Tokorozawa, Saitama, Japan; Université Joseph Fourier, France

## Abstract

The motor nerve of the bi-articular rectus femoris muscle is generally split from the femoral nerve trunk into two sub-branches just before it reaches the distal and proximal regions of the muscle. In this study, we examined whether the regional difference in muscle activities exists within the human rectus femoris muscle during maximal voluntary isometric contractions of knee extension and hip flexion. Surface electromyographic signals were recorded from the distal, middle, and proximal regions. In addition, twitch responses were evoked by stimulating the femoral nerve with supramaximal intensity. The root mean square value of electromyographic amplitude during each voluntary task was normalized to the maximal compound muscle action potential amplitude (M-wave) for each region. The electromyographic amplitudes were significantly smaller during hip flexion than during knee extension task for all regions. There was no significant difference in the normalized electromyographic amplitude during knee extension among regions within the rectus femoris muscle, whereas those were significantly smaller in the distal than in the middle and proximal regions during hip flexion task. These results indicate that the bi-articular rectus femoris muscle is differentially controlled along the longitudinal direction and that in particular the distal region of the muscle cannot be fully activated during hip flexion.

## Introduction

Some human skeletal muscles are anatomically subdivided into neuromuscular compartments, according to their architecture and/or innervation patterns [1], [2]. Previous studies have shown that in multifunctional muscles the recruitment pattern of motor units (MUs) depends on the task to which the muscle specifically contributes in mammals [3], [4], [5]. ter Haar Romeny and colleagues reported that MUs in the medial region of the long head of the biceps brachii muscle were preferably recruited during isometric forearm supination or shoulder abduction whereas MUs in lateral region were recruited mainly during isometric elbow flexion [4], [5]. It is expected that these task- and region-dependent muscle activities are observed in other muscles that are multifunctional, such as bi-articular muscles. For example, the motor nerve of the bi-articular rectus femoris (RF) muscle is generally split from the femoral nerve trunk into two sub-branches just before it reaches the distal-middle and proximal regions of the muscle [1]. With such anatomical organization in the RF, it is hypothesized that the proximal and distal regions of RF demonstrate neuromuscular activation depending on the tasks (e.g. hip flexion and knee extension). However, available data are limited to gross anatomical description [1], [6]. Therefore, this study was undertaken to compare electromyographic (EMG) activities recorded from the distal and proximal regions of the RF muscle during maximal voluntary isometric contractions (MVCs) of knee extension and hip flexion. The findings obtained in the present study can help not only in gaining insight into the functional role of the neuromuscular compartments within the RF muscle, but also in designing injury prevention and rehabilitation program for muscle strain, because theoretically, in bi-articular muscle, the activity within a muscle results in equal force at both origin and insertion but this is not necessarily the case [7].

## Methods

### Subject

Twelve healthy male subjects (27.1±3.3 years, 1.74±0.05 m, 67.3±5.4 kg; mean ± SD) participated in this study. At the time of the study, they were either sedentary or moderately active, and none had been involved in regular strength training for at least half a year. Before participation, the subjects were fully informed of the procedures as well as the purpose of the study, and gave written informed consent. This study was approved by the local ethics committee on human research, and all procedures were conducted in accordance with the Declaration of Helsinki.

### Experimental procedures

Each subject participated in familiarization and experimental sessions. Forty-eight to 72 hours before the experimental session, subjects attended a familiarization session in which they were asked to perform several isometric submaximal and maximal voluntary contractions of knee extension and hip flexion. The purpose of this session was to familiarize the subjects with the maximal voluntary torque production on a dynamometer (VTK-002, VINE, Japan) in seated position with the hip and knee flexed at 80° and 90°, respectively. In the experimental session, the subject was seated on the dynamometer, and the torso was tightly secured to the dynamometer's seat with Velcro straps. The lever arm for measurement of knee extension torque was attached 2–3 cm above the lateral malleolus, and the pad for hip flexion measurement was positioned approximately 5 cm proximal from the upper border of the patella.

**Figure 1:**
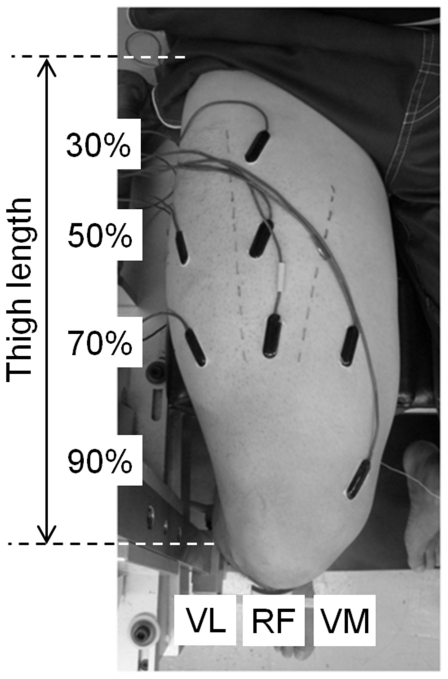
Positions of electromyogram electrodes. VL: vastus lateralis, RF: rectus femoris, VM: vastus medialis.

The surface electromyographic (EMG) signals were picked up from the vastus medialis, vastus lateralis, and rectus femoris (RF) muscles by bipolar Ag/AgCl electrodes (3 mm diameter, 10 mm inter-electrode distance), with band-pass filtering between 5 Hz and 3 kHz (gain: ×500, input impedance: >100 MΩ, CMRR: >80 dB; MEG-6116, Nihon-Kohden, Japan). The electrodes were placed over at the level of approximately 70% (proximal) and 90% (distal) of the thigh length between the greater trochanter and the lateral condyle of the femur for the vastus medialis muscle, 50% and 70% for the vastus lateralis, and 30%, 50% and 70% length for RF (Fig. 1). At all positions, in order to minimize the cross-talk from neighboring muscles, the borders between muscles were identified with the help of ultrasonography (SSD-6500, Aloka, Japan). Furthermore, it is well understood that when bipolar electrodes are used, both electrodes need to be placed either proximally or distally to the test muscles' innervation zone in order to reduce the chance that a great proportion of the signal will be lost due to phase cancellation [8], [9]. Thus, special care was taken for the electrodes' position and direction. In detail, the longitudinal orientations of the fascicles of RF were visualized with ultrasonography and the electrodes were placed along the fascicles. Then, twitch stimulation was applied to the femoral nerve and a clear biphasic M-wave in all muscles was confirmed to check whether the EMG electrodes were placed correctly. The procedure to evoke the quadriceps twitch has been described in detail elsewhere [10], [11]. Briefly, the femoral nerve was stimulated percutaneously using the cathode (2×2 cm) at the femoral triangle with supramaximal intensity (20% above maximum) of rectangular pulses of 500 µs duration which were delivered from a high-voltage stimulator (SEN-3301, Nihon Kohden, Japan). The reference electrode was placed over the left patella for all EMG measurements. The electrode placement was preceded by shaving, abrasion, and cleaning of the skin with alcohol to reduce the source impedance. After warm-up contractions, the responses to singlet for the measurement of M-wave amplitude were recorded. Thereafter, the subject performed four MVCs of knee extension or hip flexion (2 trials for each task) for approximately 3 s, with a rest period of 2 min in random order for each subject. The torque and EMG data were simultaneously recorded using a 16-bit analogue-to-digital converter (PowerLab/16SP, ADInstrument, Australia) with a sampling frequency of 4 kHz.

### Data analysis

The data from the trial in which the peak torque during MVC was higher between two contractions in the assessment for each task were adopted for analysis. EMG amplitude of each signal of the vastus medialis, vastus lateralis, and RF during knee extension or hip flexion MVC was quantified as the root-mean-square value (RMS_MVC_) over a 1 s period with a steady torque output. Regarding M-wave data, the peak-to-peak amplitude (Amp_Mwave_) and RMS values (RMS_Mwave_) during the period corresponding to the area above baseline were calculated. The RMS_MVC_ values were then normalized to Amp_Mwave_ and RMS_Mwave_ for the respective signals.

### Statistical analysis

For each muscle, separate two-way analysis of variance (ANOVA) (region×task) with repeated measures was used. When a significant interaction was observed, additional one-way ANOVAs with post hoc were performed. The significance level for all comparisons was set at P<0.05. Data are expressed as means ± SD.

## Results

**Figure 2:**
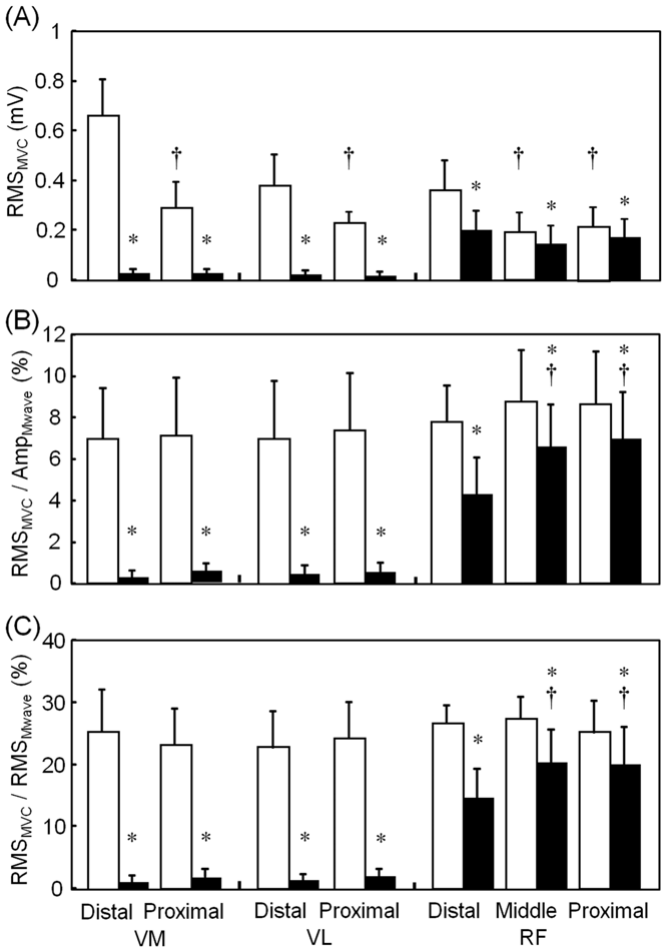
Electromyographic activities. The root-mean-square (RMS) values of electromyographic activities of the three superficial knee extensor muscles during maximal voluntary isomeric contractions of knee extension (open bar) and hip flexion (closed bar). A: absolute RMS values (RMS_MVC_), B: RMS values normalized to the peak-to-peak amplitude of the maximal compound muscle action potential of each region (RMS_MVC_/AMP_Mwave_), C: RMS values normalized to the RMS values of the maximal compound muscle action potential of reach region (RMS_MVC_/RMS_Mwave_). Results are presented as the mean ± SD (n = 12). *Significant difference (P<0.05) between the two tasks. †Significant difference (P<0.05) from distal region.

MVC torque values were 208.9±33.1 Nm and 162.2±32.7 Nm for knee extension and hip flexion, respectively. For the absolute EMG values, there were significant main effects of region and task and no significant interaction in both vastus medialis and lateralis muscles, whereas in the RF muscle there was a significant interaction between region and task (Fig. 2A). For the normalized EMG values, however, there was a significant main effect of task on RMS_MVC_/AMP_Mwave_ and RMS_MVC_/RMS_Mwave_ and no significant interaction in both vastus medialis and lateralis muscles, whereas a significant interaction was observed in RF muscle (P<0.05; Fig. 2B, 2C). Follow-up post hoc comparisons for the RF muscle revealed that both RMS_MVC_/Amp_Mwave_ and RMS_MVC_/RMS_Mwave_ were significantly smaller during hip flexion than during knee extension MVC task for all regions. The relative muscle activities of RF during hip flexion to knee extension MVC task were 55.5±17.0%, 73.7±16.1%, and 80.1±17.2% for the distal, middle, and proximal regions, respectively. Moreover, there was no significant difference in both RMS_MVC_/Amp_Mwave_ and RMS_MVC_/RMS_Mwave_ values between regions for knee extension task, whereas for hip flexion task the distal region were significantly smaller than those of the middle and proximal regions of the RF muscle for the RMS_MVC_/Amp_Mwave_ value (distal: 4.40±1.75%, middle: 6.58±2.28%, proximal: 7.02±2.65%; Fig. 2A) and for the RMS_MVC_/RMS_Mwave_ (distal: 14.8±4.6%, middle: 20.4±5.5%, proximal: 20.4±6.1%; Fig. 2B).

## Discussion

The major findings of this study were that 1) muscle activities of RF were significantly smaller during hip flexion MVC than knee extension MVC task in all regions, and that 2) there was no significant difference in muscle activities of RF during knee extension MVC among regions whereas the muscle activity of the distal region was significantly smaller than those of the middle and proximal regions during hip flexion MVC. From the present findings, it is suggested that the distal and middle-proximal neuromuscular compartments within the RF muscle exist and that individual neuromuscular compartments are separately regulated, especially during hip flexion task. It is one of the important goals in motor control researches to clarify how the central nervous system coordinates multiple neuromuscular compartments for different joint torque production. To the best of our knowledge, this is the first study to demonstrate the inhomogeneous muscle activation within RF only for hip flexion, not for knee extension task.

Before interpreting the results for EMG activities of the rectus femoris muscle, a mention should be made of the methodology used in the present study. The magnitude itself of the EMG amplitude is influenced by many electrodes' position-dependent differences in peripheral factors such as muscle membrane excitability, the number of muscle fibers within the EMG electrode recording volume, skin impedance, and the position of recording electrodes relative to the innervation zone [12], [13], [14], [15], [16] and consequently we cannot directly compare the magnitudes of the EMG amplitude between muscles and between regions within a muscle. Most recently, similarly to the present study, Watanebe et al. (2012) examined task- and region-dependent muscle activities within the RF muscle by using multi-channel surface EMG [17]. They reported that the muscle activity in the distal region during knee extension task was higher compared with that in the proximal region, and that the RF muscle was prominently activated in the proximal region during hip flexion task [17]. Their results are partially inconsistent with the present findings. In the previous study, the RMS values for each EMG signal were normalized to those obtained during MVC task. Since the muscle activation level is not necessarily uniform within a muscle even during MVC, the previous results may not accurately reflect the regionally inhomogeneous muscle activities, because of this normalization procedure. The procedure that the EMG amplitude during voluntary contraction (e.g. mean and RMS value) is normalized to the size of the maximal compound muscle action potential (M-wave) for each EMG signal can exclude the differences in peripheral influences from the interpretation of the EMG data, and enable us to compare the magnitude of central command between regions [18], [19].

The task- and region-dependent muscle activities within a muscle have been reported in other human muscles such as the triceps surae [8], biceps brachii [4], [5], [20] and trapezius muscles [21]. These task-dependent regionally inhomogeneous muscle activities have been partly explained by a clustering in limited territory of muscle fibers which belong to one motor unit [22], [23], [24]. In addition to this, Chanaud and Macpherson (1991) suggested that the inhomogeneous activities observed within the cat biceps femoris muscle during various tasks were attributed to the difference in spatial facilitation at spinal and/or supraspinal levels [23]. Furthermore, Sacco et al. (1997) demonstrated that the depressed EMG activity of the medial gastrocnemius muscle during isometric MVC of plantar flexion was observed after the selective fatigue of the lateral gastrocnemius muscle induced by surface electrical stimulation [25]. Taken together, these findings indicate a possibility that there are facilitatory connections between synergistic muscles at spinal and/or supraspinal levels, and thus in this study it is expected that net synaptic input to the motoneurons of the distal region of RF was increased by facilitation from the vasti muscles during knee extension task.

Another possible mechanism is the inhibition by activation of the synergistic muscle(s). By using the method of post-stimulus time histogram, Naito et al. (1996) reported an oligosynaptic inhibitory reflex pathway from the brachioradialis to the biceps brachii motoneurons [26]. The distribution of synaptic input is distinct across different regions within a muscle [27] and synaptic inputs from corticospinal neurons to the motoneuron pools were also segregated [28]. Therefore, it is possible that in this study the strength of the inhibition through the inhibitory reflex pathway from the hip flexion synergists like the psoas major muscle differs among regions within the RF muscle during hip flexion task. Due to paucity of information on the neural pathways in the human nervous system controlling knee extension and hip flexion, however, further investigations with more direct electrophysiological measurements such as corticospinal neurons and motoneurons excitabilities are warranted to identify the detail mechanisms responsible for the task-dependent regional difference in muscle activities within RF.

Previous studies have reported region-specific adaptations in muscle size of RF in response to resistance training [29], [30]. For example, Narici et al. [30] demonstrated that the extent of the muscle hypertrophy of RF induce by knee extension training for 60 days was greater in the distal region than in the proximal region. They proposed the difference in muscle activation during the resistance exercise as an explanation for the region-specific muscle hypertrophy. According to the present findings, although the contraction intensity and repetitions of knee extension exercise differ between the previous and present studies, other mechanism(s) would attribute to the nonuniform muscle hypertrophy of the RF muscle.

It is well known that muscle strain injuries occur more frequently in bi-articular muscles compared with mono-articular muscles, and that especially the RF muscle and the long head of the biceps femoris muscle have greater susceptibility for the injury among the thigh muscles [31], [32], [33]. The innervation pattern of these muscles has been proposed as a likely mechanism for their remarkable susceptibility to injury [32], [33], [34]. If we assume that the magnitude of neuromuscular activation and its variation within a muscle reflect forces of individual muscle components [35], [36], the results of the present study indicate nonuniform force distribution within the RF muscle, which may support the proposed explanation for the preferential occurrence of muscle strain injury in this muscle.

Despite all the precautions taken with the placement of the recording electrodes, cross-talk might have occurred as some previous studies have indicated the effect of cross-talk from other muscles on surface EMG signal of the RF muscle [37], [38]. This could constitute a potential limitation of the present results. However, miniature electrodes (3 mm diameter and 10 mm inter-electrode distance) were used in this study. Furthermore, the RMS-EMG of the vastus medialis and lateralis muscles during hip flexion tasks was slight. Therefore, we believe that the effect of the cross-talk on the results in this study was negligible, although further investigation with more direct electrophysiological approaches such as intramuscular EMG and electrical stimulation is warranted to examine the effect of cross-talk.

In conclusion, the RF muscle is activated at comparable levels along the longitudinal direction during knee extension MVC task, whereas the muscle activities are inhomogeneous within the RF muscle during hip flexion MVC, with a smaller activity in distal region compared to the middle and proximal regions. These results suggest that the central nervous system differentially controls the bi-articular RF muscle along the longitudinal direction and cannot fully activate the distal region of the muscle during hip flexion. Thus, the present findings may help in designing rehabilitation and training program for the RF muscle dysfunction due to injuries such as muscle strain.
